# County, subregional and regional phosphorus data derived from the net anthropogenic nitrogen/phosphorus inputs (NANI/NAPI) toolbox

**DOI:** 10.1016/j.dib.2019.104265

**Published:** 2019-07-25

**Authors:** Dennis P. Swaney, Robert W. Howarth

**Affiliations:** Cornell University, USA

**Keywords:** Agricultural biogeochemistry, Net anthropogenic phosphorus inputs (NAPI), Phosphorus, Phosphorus use efficiency (PUE), Regional patterns

## Abstract

The data presented here represent estimates of the phosphorus content of crop production, phosphorus use efficiency (PUE) and agricultural phosphorus inputs associated with it across the contiguous United States. Net Anthropogenic Phosphorus Input (NAPI) estimates and related data are also provided. Data are presented at county, sub-regional and regional scales. Here, subregions refer to multi-county areas delineated with the goal of obtaining more uniform reporting areas than individual counties. Regions refer to the USDA Farm Resource Regions. The data are reported for 6 agricultural census years, 1987, 1992, 1997, 2002, 2007 and 2012. Estimates of the variables were derived originally from USDA agricultural census data, US population census data, and other sources, using version 3.1 of the NANI/NAPI calculator toolbox (Hong et al., 2011).

**Table:** Specifications Table

Subject area	*Biogeochemistry, agriculture, ecology*
More specific subject area	*Phosphorus cycling, phosphorus use efficiency, net anthropogenic phosphorus inputs (NAPI)*
Type of data	*Tables, multi-page spreadsheet*
How data was acquired	*Derived from publicly available sources, including US Census of Agriculture, US population Census, and processed using the NANI/NAPI toolbox*
Data format	*Raw*
Parameters for data selection	*County level data were typically estimated from agricultural and population census information. Data were aggregated to multi-county units to achieve better spatial uniformity, and to USDA-ERS Farm Resource Regions for regional comparison.*
Parameters for data collection	*Basic data are not derived from experiments, but represent county-level estimates based on government statistics typically collected at 5-year intervals*
Data source location	*Continental United States*
Data accessibility	*Data are published with this article. Source data from which these data are derived were obtained from the NANI/NAPI toolbox, Swaney, D.P., Hong, B., and Howarth, R.W. 2018. NANI/NAPI Calculator Toolbox Version 3.1 Documentation.**http://www.eeb.cornell.edu/biogeo/nanc/nani/NANI**NAPI_Calculator_Toolbox_Version_3.1_Documentation.docx*
Related research article	*D.P. Swaney, R.W. Howarth, Phosphorus use efficiency and crop production: Patterns of regional variation in the United States, 1987–2012. . Science of the Total Environment, 685 (2019) 174–188**https://doi.org/10.1016/j.scitotenv.2019.05.228*

**Table:** 

**Value of the data** • Why are these data useful? They represent county-level to regional variation of major agricultural phosphorus flows across the US over 3 decades at 5-year intervals, and are thus useful for biogeochemical, ecological or agricultural studies of local to regional variation • Who can benefit from these data? The data should be useful to environmental research scientists interested in understanding agricultural impacts on the environment as well as patterns of agricultural production. Environmental managers at the federal, state and local level, as well as NGOs, may also find them useful. • How can these data be used for further insights and development of experiments? These data provide information on phosphorus use efficiency, surplus/deficits, and anthropogenic P inputs useful for establishing nutrient budgets at various scales, for regional comparisons, and as baseline data against which to compare impacts of alternative management options. • The data are of sufficient resolution (county and multi-county) that they may also be useful for within-region or large-watershed studies.

## 1 Data

A full list of the variables provided at county, subregional scales, together with a short description of the variables and their units are provided in Table 1, Table 2, Table 3. Details on the characteristics of the regions and subregions considered are provided in Table 1 of [5], together with maps of regions (Figure 1) and subregions (Fig. S1) in the same paper.

**Table 1:** Description of variables provided at the county-scale.

Variable Name	Units/Format	Description
FIPScode	number	Federal Information Processing Standard county code
Region Code	Text	Region Code
Region Name	Text	Region Name
State	Text	State Name
State Code	Text	State Code
County Name	Text	County Name
Subregion Code	Text	Subregion Code
County area	km^2^	County area
NAPI 1987	kg P km^−2^ yr^−1^	Net anthropogenic phosphorus inputs to counties, 1987
NAPI 1992	kg P km^−2^ yr^−1^	Net anthropogenic phosphorus inputs to counties, 1992
NAPI 1997	kg P km^−2^ yr^−1^	Net anthropogenic phosphorus inputs to counties, 1997
NAPI 2002	kg P km^−2^ yr^−1^	Net anthropogenic phosphorus inputs to counties, 2002
NAPI 2007	kg P km^−2^ yr^−1^	Net anthropogenic phosphorus inputs to counties, 2007
NAPI 2012	kg P km^−2^ yr^−1^	Net anthropogenic phosphorus inputs to counties, 2012
NFF 1987	kg P km^−2^ yr^−1^	Net food/feed phosphorus inputs to counties, 1987
NFF 1992	kg P km^−2^ yr^−1^	Net food/feed phosphorus inputs to counties, 1992
NFF 1997	kg P km^−2^ yr^−1^	Net food/feed phosphorus inputs to counties, 1997
NFF 2002	kg P km^−2^ yr^−1^	Net food/feed phosphorus inputs to counties, 2002
NFF 2007	kg P km^−2^ yr^−1^	Net food/feed phosphorus inputs to counties, 2007
NFF 2012	kg P km^−2^ yr^−1^	Net food/feed phosphorus inputs to counties, 2012
NonFEXP 1987	kg P km^−2^ yr^−1^	Nonfood P export from counties, 1987
NonFEXP 1992	kg P km^−2^ yr^−1^	Nonfood P export from counties, 1992
NonFEXP 1997	kg P km^−2^ yr^−1^	Nonfood P export from counties, 1997
NonFEXP 2002	kg P km^−2^ yr^−1^	Nonfood P export from counties, 2002
NonFEXP 2007	kg P km^−2^ yr^−1^	Nonfood P export from counties, 2007
NonFEXP 2012	kg P km^−2^ yr^−1^	Nonfood P export from counties, 2012
PFERT 1987	kg P km^−2^ yr^−1^	Total fertilizer phosphorus inputs to counties, 1987
PFERT 1992	kg P km^−2^ yr^−1^	Total fertilizer phosphorus inputs to counties, 1992
PFERT 1997	kg P km^−2^ yr^−1^	Total fertilizer phosphorus inputs to counties, 1997
PFERT 2002	kg P km^−2^ yr^−1^	Total fertilizer phosphorus inputs to counties, 2002
PFERT 2007	kg P km^−2^ yr^−1^	Total fertilizer phosphorus inputs to counties, 2007
PFERT 2012	kg P km^−2^ yr^−1^	Total fertilizer phosphorus inputs to counties, 2012
AG PFERT 1987	kg P km^−2^ yr^−1^	Agricultural fertilizer phosphorus inputs to counties, 1987
AG PFERT 1992	kg P km^−2^ yr^−1^	Agricultural fertilizer phosphorus inputs to counties, 1992
AG PFERT 1997	kg P km^−2^ yr^−1^	Agricultural fertilizer phosphorus inputs to counties, 1997
AG PFERT 2002	kg P km^−2^ yr^−1^	Agricultural fertilizer phosphorus inputs to counties, 2002
AG PFERT 2007	kg P km^−2^ yr^−1^	Agricultural fertilizer phosphorus inputs to counties, 2007
AG PFERT 2012	kg P km^−2^ yr^−1^	Agricultural fertilizer phosphorus inputs to counties, 2012
TOTAL CROPLAND AREA 1987	km^2^	Total cropland area, 1987
TOTAL CROPLAND AREA 1992	km^2^	Total cropland area, 1992
TOTAL CROPLAND AREA 1997	km^2^	Total cropland area, 1997
TOTAL CROPLAND AREA 2002	km^2^	Total cropland area, 2002
TOTAL CROPLAND AREA 2007	km^2^	Total cropland area, 2007
TOTAL CROPLAND AREA 2012	km^2^	Total cropland area, 2012
Y_P_’, CROP P/TOT AREA 1987	kg P km^−2^ yr^−1^	P content of crops per area of county, 1987
Y_P_’, CROP P/TOT AREA 1992	kg P km^−2^ yr^−1^	P content of crops per area of county, 1992
Y_P_’, CROP P/TOT AREA 1997	kg P km^−2^ yr^−1^	P content of crops per area of county, 1997
Y_P_’, CROP P/TOT AREA 2002	kg P km^−2^ yr^−1^	P content of crops per area of county, 2002
Y_P_’, CROP P/TOT AREA 2007	kg P km^−2^ yr^−1^	P content of crops per area of county, 2007
Y_P_’, CROP P/TOT AREA 2012	kg P km^−2^ yr^−1^	P content of crops per area of county, 2012
Y_P_, CROP P/CROPLAND AREA 1987	kg P km^−2^ yr^−1^*	P content of crops per total cropland area, 1987
Y_P_, CROP P/CROPLAND AREA 1992	kg P km^−2^ yr^−1^*	P content of crops per total cropland area, 1992
Y_P_, CROP P/CROPLAND AREA 1997	kg P km^−2^ yr^−1^*	P content of crops per total cropland area, 1997
Y_P_, CROP P/CROPLAND AREA 2002	kg P km^−2^ yr^−1^*	P content of crops per total cropland area, 2002
Y_P_, CROP P/CROPLAND AREA 2007	kg P km^−2^ yr^−1^*	P content of crops per total cropland area, 2007
Y_P_, CROP P/CROPLAND AREA 2012	kg P km^−2^ yr^−1^*	P content of crops per total cropland area, 2012
AG PFERT/CROPLAND AREA, 1987	kg P km^−2^ yr^−1^*	Agricultural fertilizer phosphorus inputs to counties per area of total cropland, 1987
AG PFERT/CROPLAND AREA, 1992	kg P km^−2^ yr^−1^*	Agricultural fertilizer phosphorus inputs to counties per area of total cropland, 1992
AG PFERT/CROPLAND AREA, 1997	kg P km^−2^ yr^−1^*	Agricultural fertilizer phosphorus inputs to counties per area of total cropland, 1997
AG PFERT/CROPLAND AREA, 2002	kg P km^−2^ yr^−1^*	Agricultural fertilizer phosphorus inputs to counties per area of total cropland, 2002
AG PFERT/CROPLAND AREA, 2007	kg P km^−2^ yr^−1^*	Agricultural fertilizer phosphorus inputs to counties per area of total cropland, 2007
AG PFERT/CROPLAND AREA, 2012	kg P km^−2^ yr^−1^*	Agricultural fertilizer phosphorus inputs to counties per area of total cropland, 2012
Pop, 1987	individuals	County population, 1987
Pop, 1992	individuals	County population, 1992
Pop, 1997	individuals	County population, 1997
Pop, 2002	individuals	County population, 2002
Pop, 2007	individuals	County population, 2007
Pop, 2012	individuals	County population, 2012
PEXCR/TOT AREA 1987	kg P km^−2^ yr^−1^	Livestock P excretion per total county area, 1987
PEXCR/TOT AREA 1992	kg P km^−2^ yr^−1^	Livestock P excretion per total county area, 1992
PEXCR/TOT AREA 1997	kg P km^−2^ yr^−1^	Livestock P excretion per total county area, 1997
PEXCR/TOT AREA 2002	kg P km^−2^ yr^−1^	Livestock P excretion per total county area, 2002
PEXCR/TOT AREA 2007	kg P km^−2^ yr^−1^	Livestock P excretion per total county area, 2007
PEXCR/TOT AREA 2012	kg P km^−2^ yr^−1^	Livestock P excretion per total county area, 2012
PEXCR/CROPLAND AREA 1987	kg P km^−2^ yr^−1^*	Livestock P excretion per total cropland area, 1987
PEXCR/CROPLAND AREA 1992	kg P km^−2^ yr^−1^*	Livestock P excretion per total cropland area, 1992
PEXCR/CROPLAND AREA 1997	kg P km^−2^ yr^−1^*	Livestock P excretion per total cropland area, 1997
PEXCR/CROPLAND AREA 2002	kg P km^−2^ yr^−1^*	Livestock P excretion per total cropland area, 2002
PEXCR/CROPLAND AREA 2007	kg P km^−2^ yr^−1^*	Livestock P excretion per total cropland area, 2007
PEXCR/CROPLAND AREA 2012	kg P km^−2^ yr^−1^*	Livestock P excretion per total cropland area, 2012
I_P_, 1987	kg P km^−2^ yr^−1^*	Total ag P inputs per cropland area, 1987
I_P_, 1992	kg P km^−2^ yr^−1^*	Total ag P inputs per cropland area, 1992
I_P_, 1997	kg P km^−2^ yr^−1^*	Total ag P inputs per cropland area, 1997
I_P_, 2002	kg P km^−2^ yr^−1^*	Total ag P inputs per cropland area, 2002
I_P_, 2007	kg P km^−2^ yr^−1^*	Total ag P inputs per cropland area, 2007
I_P_, 2012	kg P km^−2^ yr^−1^*	Total ag P inputs per cropland area, 2012
I_P_’, 1987	kg P km^−2^ yr^−1^	Total ag P inputs per county area, 1987
I_P_’, 1992	kg P km^−2^ yr^−1^	Total ag P inputs per county area, 1992
I_P_’, 1997	kg P km^−2^ yr^−1^	Total ag P inputs per county area, 1997
I_P_’, 2002	kg P km^−2^ yr^−1^	Total ag P inputs per county area, 2002
I_P_’, 2007	kg P km^−2^ yr^−1^	Total ag P inputs per county area, 2007
I_P_’, 2012	kg P km^−2^ yr^−1^	Total ag P inputs per county area, 2012
PUE, 1987	dimensionless	Phosphorus Use Efficiency, Y/I, 1987
PUE, 1992	dimensionless	Phosphorus Use Efficiency, Y/I, 1992
PUE, 1997	dimensionless	Phosphorus Use Efficiency, Y/I, 1997
PUE, 2002	dimensionless	Phosphorus Use Efficiency, Y/I, 2002
PUE, 2007	dimensionless	Phosphorus Use Efficiency, Y/I, 2007
PUE, 2012	dimensionless	Phosphorus Use Efficiency, Y/I, 2012
PFERT%, 1987	%	Ag P fertilizer as % of total county P input, 1987
PFERT%, 1992	%	Ag P fertilizer as % of total county P input, 1992
PFERT%, 1997	%	Ag P fertilizer as % of total county P input, 1997
PFERT%, 2002	%	Ag P fertilizer as % of total county P input, 2002
PFERT%, 2007	%	Ag P fertilizer as % of total county P input, 2007
PFERT%, 2012	%	Ag P fertilizer as % of total county P input, 2012
PEXCR%, 1987	%	Livestock P excr. % of total county P input, 1987
PEXCR%, 1992	%	Livestock P excr. % of total county P input, 1992
PEXCR%, 1997	%	Livestock P excr. % of total county P input, 1997
PEXCR%, 2002	%	Livestock P excr. % of total county P input, 2002
PEXCR%, 2007	%	Livestock P excr. % of total county P input, 2007
PEXCR%, 2012	%	Livestock P excr. % of total county P input, 2012
LP 1987**	kg P km^−2^ yr^−1^	Production (P) of livestock products in counties, 1987
LP 1992**	kg P km^−2^ yr^−1^	Production (P) of livestock products in counties, 1992
LP 1997**	kg P km^−2^ yr^−1^	Production (P) of livestock products in counties, 1997
LP 2002**	kg P km^−2^ yr^−1^	Production (P) of livestock products in counties, 2002
LP 2007**	kg P km^−2^ yr^−1^	Production (P) of livestock products in counties, 2007
LP 2012**	kg P km^−2^ yr^−1^	Production (P) of livestock products in counties, 2012
NONCROPAND PAST AREA 1987	km^2^	County level noncropland pasture area, 1987
NONCROPAND PAST AREA 1992	km^2^	County level noncropland pasture area, 1992
NONCROPAND PAST AREA 1997	km^2^	County level noncropland pasture area, 1997
NONCROPAND PAST AREA 2002	km^2^	County level noncropland pasture area, 2002
NONCROPAND PAST AREA 2007	km^2^	County level noncropland pasture area, 2007
NONCROPAND PAST AREA 2012	km^2^	County level noncropland pasture area, 2012
fg 1987	fraction	County level fraction of manure P from grazers, 1987
fg 1992	fraction	County level fraction of manure P from grazers, 1992
fg 1997	fraction	County level fraction of manure P from grazers, 1997
fg 2002	fraction	County level fraction of manure P from grazers, 2002
fg 2007	fraction	County level fraction of manure P from grazers, 2007
fg 2012	fraction	County level fraction of manure P from grazers, 2012
fp 1987	fraction	County level fraction of noncropland pasture area relative to total cropland + noncropland pasture, 1987
fp 1992	fraction	County level fraction of noncropland pasture area relative to total cropland + noncropland pasture, 1992
fp 1997	fraction	County level fraction of noncropland pasture area relative to total cropland + noncropland pasture, 1997
fp 2002	fraction	County level fraction of noncropland pasture area relative to total cropland + noncropland pasture, 2002
fp 2007	fraction	County level fraction of noncropland pasture area relative to total cropland + noncropland pasture, 2007
fp 2012	fraction	County level fraction of noncropland pasture area relative to total cropland + noncropland pasture, 2012
1-fp *fg 1987	fraction	Estimated county level proportion of manure P available to cropland after noncropland pasture losses, 1987
1-fp *fg 1992	fraction	Estimated county level proportion of manure P available to cropland after noncropland pasture losses, 1992
1-fp *fg 1997	fraction	Estimated county level proportion of manure P available to cropland after noncropland pasture losses, 1997
1-fp *fg 2002	fraction	Estimated county level proportion of manure P available to cropland after noncropland pasture losses, 2002
1-fp *fg 2007	fraction	Estimated county level proportion of manure P available to cropland after noncropland pasture losses, 2007
1-fp*fg 2012	fraction	Estimated county level proportion of manure P available to cropland after noncropland pasture losses, 2012

*Indicates cropland area basis instead of total area basis.

**Beef & veal, pork, poultry, lamb, eggs, milk.

**Table 2:** Description of variables provided at the subregional scale.

Variable Name	Units/Format	Description
Subregion Code	Text	Subregion Code
Region Name	Text	Region Name
Region Code	Text	Region Code
Number of counties	Integer	Number of counties in subregion
Subregion area	km^2^	Area of subregion
NAPI 1987	kg P km^−2^ yr^−1^	Net anthropogenic phosphorus inputs to subregions, 1987
NAPI 1992	kg P km^−2^ yr^−1^	Net anthropogenic phosphorus inputs to subregions, 1992
NAPI 1997	kg P km^−2^ yr^−1^	Net anthropogenic phosphorus inputs to subregions, 1997
NAPI 2002	kg P km^−2^ yr^−1^	Net anthropogenic phosphorus inputs to subregions, 2002
NAPI 2007	kg P km^−2^ yr^−1^	Net anthropogenic phosphorus inputs to subregions, 2007
NAPI 2012	kg P km^−2^ yr^−1^	Net anthropogenic phosphorus inputs to subregions, 2012
NFF 1987	kg P km^−2^ yr^−1^	Net food/feed phosphorus inputs to subregions, 1987
NFF 1992	kg P km^−2^ yr^−1^	Net food/feed phosphorus inputs to subregions, 1992
NFF 1997	kg P km^−2^ yr^−1^	Net food/feed phosphorus inputs to subregions, 1997
NFF 2002	kg P km^−2^ yr^−1^	Net food/feed phosphorus inputs to subregions, 2002
NFF 2007	kg P km^−2^ yr^−1^	Net food/feed phosphorus inputs to subregions, 2007
NFF 2012	kg P km^−2^ yr^−1^	Net food/feed phosphorus inputs to subregions, 2012
NonFEXP 1987	kg P km^−2^ yr^−1^	Nonfood P export from subregions, 1987
NonFEXP 1992	kg P km^−2^ yr^−1^	Nonfood P export from subregions, 1992
NonFEXP 1997	kg P km^−2^ yr^−1^	Nonfood P export from subregions, 1997
NonFEXP 2002	kg P km^−2^ yr^−1^	Nonfood P export from subregions, 2002
NonFEXP 2007	kg P km^−2^ yr^−1^	Nonfood P export from subregions, 2007
NonFEXP 2012	kg P km^−2^ yr^−1^	Nonfood P export from subregions, 2012
PFERT 1987	kg P km^−2^ yr^−1^	Total fertilizer phosphorus inputs to subregions, 1987
PFERT 1992	kg P km^−2^ yr^−1^	Total fertilizer phosphorus inputs to subregions, 1992
PFERT 1997	kg P km^−2^ yr^−1^	Total fertilizer phosphorus inputs to subregions, 1997
PFERT 2002	kg P km^−2^ yr^−1^	Total fertilizer phosphorus inputs to subregions, 2002
PFERT 2007	kg P km^−2^ yr^−1^	Total fertilizer phosphorus inputs to subregions, 2007
PFERT 2012	kg P km^−2^ yr^−1^	Total fertilizer phosphorus inputs to subregions, 2012
AG PFERT 1987	kg P km^−2^ yr^−1^	Agricultural fertilizer phosphorus inputs to subregions, 1987
AG PFERT 1992	kg P km^−2^ yr^−1^	Agricultural fertilizer phosphorus inputs to subregions, 1992
AG PFERT 1997	kg P km^−2^ yr^−1^	Agricultural fertilizer phosphorus inputs to subregions, 1997
AG PFERT 2002	kg P km^−2^ yr^−1^	Agricultural fertilizer phosphorus inputs to subregions, 2002
AG PFERT 2007	kg P km^−2^ yr^−1^	Agricultural fertilizer phosphorus inputs to subregions, 2007
AG PFERT 2012	kg P km^−2^ yr^−1^	Agricultural fertilizer phosphorus inputs to subregions, 2012
Subregion area	km^2^	Subregion area
TOTAL CROPLAND AREA 1987	km^2^	Total subregional cropland area, 1987
TOTAL CROPLAND AREA 1992	km^2^	Total subregional cropland area, 1992
TOTAL CROPLAND AREA 1997	km^2^	Total subregional cropland area, 1997
TOTAL CROPLAND AREA 2002	km^2^	Total subregional cropland area, 2002
TOTAL CROPLAND AREA 2007	km^2^	Total subregional cropland area, 2007
TOTAL CROPLAND AREA 2012	km^2^	Total subregional cropland area, 2012
Y_P_’, CROP P/TOTAL AREA 1987	kg P km^−2^ yr^−1^	P content of crops per area of subregion, 1987
Y_P_’, CROP P/TOTAL AREA 1992	kg P km^−2^ yr^−1^	P content of crops per area of subregion, 1992
Y_P_’, CROP P/TOTAL AREA 1997	kg P km^−2^ yr^−1^	P content of crops per area of subregion, 1997
Y_P_’, CROP P/TOTAL AREA 2002	kg P km^−2^ yr^−1^	P content of crops per area of subregion, 2002
Y_P_’, CROP P/TOTAL AREA 2007	kg P km^−2^ yr^−1^	P content of crops per area of subregion, 2007
Y_P_’, CROP P/TOTAL AREA 2012	kg P km^−2^ yr^−1^	P content of crops per area of subregion, 2012
Y_P_, CROP P/CROPLAND AREA 1987	kg P km^−2^ yr^−1^*	P content of crops per subregional cropland area, 1987
Y_P_, CROP P/CROPLAND AREA 1992	kg P km^−2^ yr^−1^*	P content of crops per subregional cropland area, 1992
Y_P_, CROP P/CROPLAND AREA 1997	kg P km^−2^ yr^−1^*	P content of crops per subregional cropland area, 1997
Y_P_, CROP P/CROPLAND AREA 2002	kg P km^−2^ yr^−1^*	P content of crops per subregional cropland area, 2002
Y_P_, CROP P/CROPLAND AREA 2007	kg P km^−2^ yr^−1^*	P content of crops per subregional cropland area, 2007
Y_P_, CROP P/CROPLAND AREA 2012	kg P km^−2^ yr^−1^*	P content of crops per subregional cropland area, 2012
AG PFERT/CROPLAND AREA, 1987	kg P km^−2^ yr^−1^*	Agricultural fertilizer phosphorus inputs to subregions per area of subregional cropland, 1987
AG PFERT/CROPLAND AREA, 1992	kg P km^−2^ yr^−1^*	Agricultural fertilizer phosphorus inputs to subregions per area of subregional cropland, 1992
AG PFERT/CROPLAND AREA, 1997	kg P km^−2^ yr^−1^*	Agricultural fertilizer phosphorus inputs to subregions per area of subregional cropland, 1997
AG PFERT/CROPLAND AREA, 2002	kg P km^−2^ yr^−1^*	Agricultural fertilizer phosphorus inputs to subregions per area of subregional cropland, 2002
AG PFERT/CROPLAND AREA, 2007	kg P km^−2^ yr^−1^*	Agricultural fertilizer phosphorus inputs to subregions per area of subregional cropland, 2007
AG PFERT/CROPLAND AREA, 2012	kg P km^−2^ yr^−1^*	Agricultural fertilizer phosphorus inputs to subregions per area of subregional cropland, 2012
Pop, 1987	individuals	Subregional population, 1987
Pop, 1992	individuals	Subregional population, 1992
Pop, 1997	individuals	Subregional population, 1997
Pop, 2002	individuals	Subregional population, 2002
Pop, 2007	individuals	Subregional population, 2007
Pop, 2012	individuals	Subregional population, 2012
PEXCR/TOT AREA 1987	kg P km^−2^ yr^−1^	Livestock P excretion per total subregion area, 1987
PEXCR/TOT AREA 1992	kg P km^−2^ yr^−1^	Livestock P excretion per total subregion area, 1992
PEXCR/TOT AREA 1997	kg P km^−2^ yr^−1^	Livestock P excretion per total subregion area, 1997
PEXCR/TOT AREA 2002	kg P km^−2^ yr^−1^	Livestock P excretion per total subregion area, 2002
PEXCR/TOT AREA 2007	kg P km^−2^ yr^−1^	Livestock P excretion per total subregion area, 2007
PEXCR/TOT AREA 2012	kg P km^−2^ yr^−1^	Livestock P excretion per total subregion area, 2012
PEXCR/CROPLAND AREA 1987	kg P km^−2^ yr^−1^*	Livestock P excretion per subregional cropland area, 1987
PEXCR/CROPLAND AREA 1992	kg P km^−2^ yr^−1^*	Livestock P excretion per subregional cropland area, 1992
PEXCR/CROPLAND AREA 1997	kg P km^−2^ yr^−1^*	Livestock P excretion per subregional cropland area, 1997
PEXCR/CROPLAND AREA 2002	kg P km^−2^ yr^−1^*	Livestock P excretion per subregional cropland area, 2002
PEXCR/CROPLAND AREA 2007	kg P km^−2^ yr^−1^*	Livestock P excretion per subregional cropland area, 2007
PEXCR/CROPLAND AREA 2012	kg P km^−2^ yr^−1^*	Livestock P excretion per subregional cropland area, 2012
I_P_, 1987	kg P km^−2^ yr^−1^*	Total ag P inputs per cropland area, 1987
I_P_, 1992	kg P km^−2^ yr^−1^*	Total ag P inputs per cropland area, 1992
I_P_, 1997	kg P km^−2^ yr^−1^*	Total ag P inputs per cropland area, 1997
I_P_, 2002	kg P km^−2^ yr^−1^*	Total ag P inputs per cropland area, 2002
I_P_, 2007	kg P km^−2^ yr^−1^*	Total ag P inputs per cropland area, 2007
I_P_, 2012	kg P km^−2^ yr^−1^*	Total ag P inputs per cropland area, 2012
I_P_’, 1987	kg P km^−2^ yr^−1^	Total ag P inputs per subregion area, 1987
I_P_’, 1992	kg P km^−2^ yr^−1^	Total ag P inputs per subregion area, 1992
I_P_’, 1997	kg P km^−2^ yr^−1^	Total ag P inputs per subregion area, 1997
I_P_’, 2002	kg P km^−2^ yr^−1^	Total ag P inputs per subregion area, 2002
I_P_’, 2007	kg P km^−2^ yr^−1^	Total ag P inputs per subregion area, 2007
I_P_’, 2012	kg P km^−2^ yr^−1^	Total ag P inputs per subregion area, 2012
PUE, 1987	%	Phosphorus Use Efficiency, Y/I, 1987
PUE, 1992	%	Phosphorus Use Efficiency, Y/I, 1992
PUE, 1997	%	Phosphorus Use Efficiency, Y/I, 1997
PUE, 2002	%	Phosphorus Use Efficiency, Y/I, 2002
PUE, 2007	%	Phosphorus Use Efficiency, Y/I, 2007
PUE, 2012	%	Phosphorus Use Efficiency, Y/I, 2012
PFERT%, 1987	%	Ag P fertilizer as % of total subregional P input, 1987
PFERT%, 1992	%	Ag P fertilizer as % of total subregional P input, 1992
PFERT%, 1997	%	Ag P fertilizer as % of total subregional P input, 1997
PFERT%, 2002	%	Ag P fertilizer as % of total subregional P input, 2002
PFERT%, 2007	%	Ag P fertilizer as % of total subregional P input, 2007
PFERT%, 2012	%	Ag P fertilizer as % of total subregional P input, 2012
PEXCR%, 1987	%	Livestock P excr. % of total subregional P input, 1987
PEXCR%, 1992	%	Livestock P excr. % of total subregional P input, 1992
PEXCR%, 1997	%	Livestock P excr. % of total subregional P input, 1997
PEXCR%, 2002	%	Livestock P excr. % of total subregional P input, 2002
PEXCR%, 2007	%	Livestock P excr. % of total subregional P input, 2007
PEXCR%, 2012	%	Livestock P excr. % of total subregional P input, 2012
LP 1987**	kg P km^−2^ yr^−1^	Production (P) of livestock products in subregions, 1987
LP 1992**	kg P km^−2^ yr^−1^	Production (P) of livestock products in subregions, 1992
LP 1997**	kg P km^−2^ yr^−1^	Production (P) of livestock products in subregions, 1997
LP 2002**	kg P km^−2^ yr^−1^	Production (P) of livestock products in subregions, 2002
LP 2007**	kg P km^−2^ yr^−1^	Production (P) of livestock products in subregions, 2007
LP 2012**	kg P km^−2^ yr^−1^	Production (P) of livestock products in subregions, 2012
NONCROPAND PAST AREA 1987	km^2^	Subregional noncropland pasture area, 1987
NONCROPAND PAST AREA 1992	km^2^	Subregional noncropland pasture area, 1992
NONCROPAND PAST AREA 1997	km^2^	Subregional noncropland pasture area, 1997
NONCROPAND PAST AREA 2002	km^2^	Subregional noncropland pasture area, 2002
NONCROPAND PAST AREA 2007	km^2^	Subregional noncropland pasture area, 2007
NONCROPAND PAST AREA 2012	km^2^	Subregional noncropland pasture area, 2012
fg 1987	fraction	Subregional fraction of manure P from grazers, 1987
fg 1992	fraction	Subregional fraction of manure P from grazers, 1992
fg 1997	fraction	Subregional fraction of manure P from grazers, 1997
fg 2002	fraction	Subregional fraction of manure P from grazers, 2002
fg 2007	fraction	Subregional fraction of manure P from grazers, 2007
fg 2012	fraction	Subregional fraction of manure P from grazers, 2012
fp 1987	fraction	Subregional fraction of noncropland pasture area relative to total cropland + noncropland pasture, 1987
fp 1992	fraction	Subregional fraction of noncropland pasture area relative to total cropland + noncropland pasture, 1992
fp 1997	fraction	Subregional fraction of noncropland pasture area relative to total cropland + noncropland pasture, 1997
fp 2002	fraction	Subregional fraction of noncropland pasture area relative to total cropland + noncropland pasture, 2002
fp 2007	fraction	Subregional fraction of noncropland pasture area relative to total cropland + noncropland pasture, 2007
fp 2012	fraction	Subregional fraction of noncropland pasture area relative to total cropland + noncropland pasture, 2012
1-fp *fg 1987	fraction	Estimated subregional proportion of manure P available to cropland after noncropland pasture losses, 1987
1-fp *fg 1992	fraction	Estimated subregional proportion of manure P available to cropland after noncropland pasture losses, 1992
1-fp *fg 1997	fraction	Estimated subregional proportion of manure P available to cropland after noncropland pasture losses, 1997
1-fp *fg 2002	fraction	Estimated subregional proportion of manure P available to cropland after noncropland pasture losses, 2002
1-fp *fg 2007	fraction	Estimated subregional proportion of manure P available to cropland after noncropland pasture losses, 2007
1-fp*fg 2012	fraction	Estimated subregional proportion of manure P available to cropland after noncropland pasture losses, 2012

*Indicates cropland area basis instead of total area basis.

**Beef & veal, pork, poultry, lamb, eggs, milk.

**Table 3:** Description of variables provided at the regional scale.

Variable Name	Units/Format	Description
Region Name	Text	Region Name
Region Code	Text	Region Code
Region Area	km^2^	Area of region
NAPI 1987	kg P km^−2^ yr^−1^	Net anthropogenic phosphorus inputs to regions, 1987
NAPI 1992	kg P km^−2^ yr^−1^	Net anthropogenic phosphorus inputs to regions, 1992
NAPI 1997	kg P km^−2^ yr^−1^	Net anthropogenic phosphorus inputs to regions, 1997
NAPI 2002	kg P km^−2^ yr^−1^	Net anthropogenic phosphorus inputs to regions, 2002
NAPI 2007	kg P km^−2^ yr^−1^	Net anthropogenic phosphorus inputs to regions, 2007
NAPI 2012	kg P km^−2^ yr^−1^	Net anthropogenic phosphorus inputs to regions, 2012
NFF 1987	kg P km^−2^ yr^−1^	Net food/feed phosphorus inputs to regions, 1987
NFF 1992	kg P km^−2^ yr^−1^	Net food/feed phosphorus inputs to regions, 1992
NFF 1997	kg P km^−2^ yr^−1^	Net food/feed phosphorus inputs to regions, 1997
NFF 2002	kg P km^−2^ yr^−1^	Net food/feed phosphorus inputs to regions, 2002
NFF 2007	kg P km^−2^ yr^−1^	Net food/feed phosphorus inputs to regions, 2007
NFF 2012	kg P km^−2^ yr^−1^	Net food/feed phosphorus inputs to regions, 2012
NonFEXP 1987	kg P km^−2^ yr^−1^	Nonfood P export from regions, 1987
NonFEXP 1992	kg P km^−2^ yr^−1^	Nonfood P export from regions, 1992
NonFEXP 1997	kg P km^−2^ yr^−1^	Nonfood P export from regions, 1997
NonFEXP 2002	kg P km^−2^ yr^−1^	Nonfood P export from regions, 2002
NonFEXP 2007	kg P km^−2^ yr^−1^	Nonfood P export from regions, 2007
NonFEXP 2012	kg P km^−2^ yr^−1^	Nonfood P export from regions, 2012
PFERT 1987	kg P km^−2^ yr^−1^	Total fertilizer phosphorus inputs to regions, 1987
PFERT 1992	kg P km^−2^ yr^−1^	Total fertilizer phosphorus inputs to regions, 1992
PFERT 1997	kg P km^−2^ yr^−1^	Total fertilizer phosphorus inputs to regions, 1997
PFERT 2002	kg P km^−2^ yr^−1^	Total fertilizer phosphorus inputs to regions, 2002
PFERT 2007	kg P km^−2^ yr^−1^	Total fertilizer phosphorus inputs to regions, 2007
PFERT 2012	kg P km^−2^ yr^−1^	Total fertilizer phosphorus inputs to regions, 2012
AG PFERT 1987	kg P km^−2^ yr^−1^	Agricultural fertilizer phosphorus inputs to regions, 1987
AG PFERT 1992	kg P km^−2^ yr^−1^	Agricultural fertilizer phosphorus inputs to regions, 1992
AG PFERT 1997	kg P km^−2^ yr^−1^	Agricultural fertilizer phosphorus inputs to regions, 1997
AG PFERT 2002	kg P km^−2^ yr^−1^	Agricultural fertilizer phosphorus inputs to regions, 2002
AG PFERT 2007	kg P km^−2^ yr^−1^	Agricultural fertilizer phosphorus inputs to regions, 2007
AG PFERT 2012	kg P km^−2^ yr^−1^	Agricultural fertilizer phosphorus inputs to regions, 2012
region area	km^2^	region area
TOTAL CROPLAND AREA 1987	km^2^	Total regional cropland area, 1987
TOTAL CROPLAND AREA 1992	km^2^	Total regional cropland area, 1992
TOTAL CROPLAND AREA 1997	km^2^	Total regional cropland area, 1997
TOTAL CROPLAND AREA 2002	km^2^	Total regional cropland area, 2002
TOTAL CROPLAND AREA 2007	km^2^	Total regional cropland area, 2007
TOTAL CROPLAND AREA 2012	km^2^	Total regional cropland area, 2012
Y_P_’, CROP P/TOT AREA 1987	kg P km^−2^ yr^−1^	P content of crops per area of region, 1987
Y_P_’, CROP P/TOT AREA 1992	kg P km^−2^ yr^−1^	P content of crops per area of region, 1992
Y_P_’, CROP P/TOT AREA 1997	kg P km^−2^ yr^−1^	P content of crops per area of region, 1997
Y_P_’, CROP P/TOT AREA 2002	kg P km^−2^ yr^−1^	P content of crops per area of region, 2002
Y_P_’, CROP P/TOT AREA 2007	kg P km^−2^ yr^−1^	P content of crops per area of region, 2007
Y_P_’, CROP P/TOT AREA 2012	kg P km^−2^ yr^−1^	P content of crops per area of region, 2012
Y_P_, CROP P/CROPLAND AREA 1987	kg P km^−2^ yr^−1^*	P content of crops per regional cropland area, 1987
Y_P_, CROP P/CROPLAND AREA 1992	kg P km^−2^ yr^−1^*	P content of crops per regional cropland area, 1992
Y_P_, CROP P/CROPLAND AREA 1997	kg P km^−2^ yr^−1^*	P content of crops per regional cropland area, 1997
Y_P_, CROP P/CROPLAND AREA 2002	kg P km^−2^ yr^−1^*	P content of crops per regional cropland area, 2002
Y_P_, CROP P/CROPLAND AREA 2007	kg P km^−2^ yr^−1^*	P content of crops per regional cropland area, 2007
Y_P_, CROP P/CROPLAND AREA 2012	kg P km^−2^ yr^−1^*	P content of crops per regional cropland area, 2012
AG PFERT/CROPLAND AREA, 1987	kg P km^−2^ yr^−1^*	Agricultural fertilizer phosphorus inputs to regions per area of regional cropland, 1987
AG PFERT/CROPLAND AREA, 1992	kg P km^−2^ yr^−1^*	Agricultural fertilizer phosphorus inputs to regions per area of regional cropland, 1992
AG PFERT/CROPLAND AREA, 1997	kg P km^−2^ yr^−1^*	Agricultural fertilizer phosphorus inputs to regions per area of regional cropland, 1997
AG PFERT/CROPLAND AREA, 2002	kg P km^−2^ yr^−1^*	Agricultural fertilizer phosphorus inputs to regions per area of regional cropland, 2002
AG PFERT/CROPLAND AREA, 2007	kg P km^−2^ yr^−1^*	Agricultural fertilizer phosphorus inputs to regions per area of regional cropland, 2007
AG PFERT/CROPLAND AREA, 2012	kg P km^−2^ yr^−1^*	Agricultural fertilizer phosphorus inputs to regions per area of regional cropland, 2012
Pop, 1987	individuals	Regional population, 1987
Pop, 1992	individuals	Regional population, 1992
Pop, 1997	individuals	Regional population, 1997
Pop, 2002	individuals	Regional population, 2002
Pop, 2007	individuals	Regional population, 2007
Pop, 2012	individuals	Regional population, 2012
PEXCR/TOT AREA 1987	kg P km-^2^ yr-^1^	Livestock P excretion per total region area, 1987
PEXCR/TOT AREA 1992	kg P km-2 yr-1	Livestock P excretion per total region area, 1992
PEXCR/TOT AREA 1997	kg P km-2 yr-1	Livestock P excretion per total region area, 1997
PEXCR/TOT AREA 2002	kg P km-2 yr-1	Livestock P excretion per total region area, 2002
PEXCR/TOT AREA 2007	kg P km-2 yr-1	Livestock P excretion per total region area, 2007
PEXCR/TOT AREA 2012	kg P km-2 yr-1	Livestock P excretion per total region area, 2012
PEXCR/CROPLAND AREA 1987	kg P km-2 yr-1*	Livestock P excretion per regional cropland area, 1987
PEXCR/CROPLAND AREA 1992	kg P km-2 yr-1*	Livestock P excretion per regional cropland area, 1992
PEXCR/CROPLAND AREA 1997	kg P km-2 yr-1*	Livestock P excretion per regional cropland area, 1997
PEXCR/CROPLAND AREA 2002	kg P km-2 yr-1*	Livestock P excretion per regional cropland area, 2002
PEXCR/CROPLAND AREA 2007	kg P km-2 yr-1*	Livestock P excretion per regional cropland area, 2007
PEXCR/CROPLAND AREA 2012	kg P km-2 yr-1*	Livestock P excretion per regional cropland area, 2012
I_P_, 1987	kg P km-2 yr-1*	Total ag P inputs per cropland area, 1987
I_P_, 1992	kg P km-2 yr-1*	Total ag P inputs per cropland area, 1992
I_P_, 1997	kg P km-2 yr-1*	Total ag P inputs per cropland area, 1997
I_P_, 2002	kg P km-2 yr-1*	Total ag P inputs per cropland area, 2002
I_P_, 2007	kg P km-2 yr-1*	Total ag P inputs per cropland area, 2007
I_P_, 2012	kg P km-2 yr-1*	Total ag P inputs per cropland area, 2012
I_P_’, 1987	kg P km-2 yr-1	Total ag P inputs per regional area, 1987
I_P_’, 1992	kg P km-2 yr-1	Total ag P inputs per regional area, 1992
I_P_’, 1997	kg P km-2 yr-1	Total ag P inputs per regional area, 1997
I_P_’, 2002	kg P km-2 yr-1	Total ag P inputs per regional area, 2002
I_P_’, 2007	kg P km-2 yr-1	Total ag P inputs per regional area, 2007
I_P_’, 2012	kg P km-2 yr-1	Total ag P inputs per regional area, 2012
PUE, 1987	%	Phosphorus Use Efficiency, Y/I, 1987
PUE, 1992	%	Phosphorus Use Efficiency, Y/I, 1992
PUE, 1997	%	Phosphorus Use Efficiency, Y/I, 1997
PUE, 2002	%	Phosphorus Use Efficiency, Y/I, 2002
PUE, 2007	%	Phosphorus Use Efficiency, Y/I, 2007
PUE, 2012	%	Phosphorus Use Efficiency, Y/I, 2012
S_P_, 1987	kg P km^−2^ yr^−1^*	Cropland P surplus/deficit, 1987
S_P_, 1992	kg P km^−2^ yr^−1^*	Cropland P surplus/deficit, 1992
S_P_, 1997	kg P km^−2^ yr^−1^*	Cropland P surplus/deficit, 1997
S_P_, 2002	kg P km^−2^ yr^−1^*	Cropland P surplus/deficit, 2002
S_P_, 2007	kg P km^−2^ yr^−1^*	Cropland P surplus/deficit, 2007
S_P_, 2012	kg P km^−2^ yr^−1^*	Cropland P surplus/deficit, 2012
PFERT%, 1987	%	Ag P fertilizer as % of total regional P input, 1987
PFERT%, 1992	%	Ag P fertilizer as % of total regional P input, 1992
PFERT%, 1997	%	Ag P fertilizer as % of total regional P input, 1997
PFERT%, 2002	%	Ag P fertilizer as % of total regional P input, 2002
PFERT%, 2007	%	Ag P fertilizer as % of total regional P input, 2007
PFERT%, 2012	%	Ag P fertilizer as % of total regional P input, 2012
PEXCR%, 1987	%	Livestock P excr. % of total regional P input, 1987
PEXCR%, 1992	%	Livestock P excr. % of total regional P input, 1992
PEXCR%, 1997	%	Livestock P excr. % of total regional P input, 1997
PEXCR%, 2002	%	Livestock P excr. % of total regional P input, 2002
PEXCR%, 2007	%	Livestock P excr. % of total regional P input, 2007
PEXCR%, 2012	%	Livestock P excr. % of total regional P input, 2012
LP 1987**	kg P km^−2^ yr^−1^	Production (P) of livestock products in regions, 1987
LP 1992**	kg P km^−2^ yr^−1^	Production (P) of livestock products in regions, 1992
LP 1997**	kg P km^−2^ yr^−1^	Production (P) of livestock products in regions, 1997
LP 2002**	kg P km^−2^ yr^−1^	Production (P) of livestock products in regions, 2002
LP 2007**	kg P km^−2^ yr^−1^	Production (P) of livestock products in regions, 2007
LP 2012**	kg P km^−2^ yr^−1^	Production (P) of livestock products in regions, 2012
NONCROPAND PAST AREA 1987	km^2^	Regional noncropland pasture area, 1987
NONCROPAND PAST AREA 1992	km^2^	Regional noncropland pasture area, 1992
NONCROPAND PAST AREA 1997	km^2^	Regional noncropland pasture area, 1997
NONCROPAND PAST AREA 2002	km^2^	Regional noncropland pasture area, 2002
NONCROPAND PAST AREA 2007	km^2^	Regional noncropland pasture area, 2007
NONCROPAND PAST AREA 2012	km^2^	Regional noncropland pasture area, 2012
fg 1987	fraction	Regional fraction of manure P from grazers, 1987
fg 1992	fraction	Regional fraction of manure P from grazers, 1992
fg 1997	fraction	Regional fraction of manure P from grazers, 1997
fg 2002	fraction	Regional fraction of manure P from grazers, 2002
fg 2007	fraction	Regional fraction of manure P from grazers, 2007
fg 2012	fraction	Regional fraction of manure P from grazers, 2012
fp 1987	fraction	Regional fraction of noncropland pasture area relative to total cropland + noncropland pasture, 1987
fp 1992	fraction	Regional fraction of noncropland pasture area relative to total cropland + noncropland pasture, 1992
fp 1997	fraction	Regional fraction of noncropland pasture area relative to total cropland + noncropland pasture, 1997
fp 2002	fraction	Regional fraction of noncropland pasture area relative to total cropland + noncropland pasture, 2002
fp 2007	fraction	Regional fraction of noncropland pasture area relative to total cropland + noncropland pasture, 2007
fp 2012	fraction	Regional fraction of noncropland pasture area relative to total cropland + noncropland pasture, 2012
1-fp *fg 1987	fraction	Estimated regional proportion of manure P available to cropland after noncropland pasture losses, 1987
1-fp *fg 1992	fraction	Estimated regional proportion of manure P available to cropland after noncropland pasture losses, 1992
1-fp *fg 1997	fraction	Estimated regional proportion of manure P available to cropland after noncropland pasture losses, 1997
1-fp *fg 2002	fraction	Estimated regional proportion of manure P available to cropland after noncropland pasture losses, 2002
1-fp *fg 2007	fraction	Estimated regional proportion of manure P available to cropland after noncropland pasture losses, 2007
1-fp*fg 2012	fraction	Estimated regional proportion of manure P available to cropland after noncropland pasture losses, 2012

*Indicates cropland area basis instead of total area basis.

**Beef & veal, pork, poultry, lamb, eggs, milk.

## 2 Experimental design, materials, and methods

Briefly, the data contained in the spreadsheets represent components of Net Anthropogenic Phosphorus Input (NAPI) and ancillary data obtained and postprocessed from these data using simple spreadsheet calculations to estimate variables used in phosphorus use efficiency estimates at county, subregional and regional scales across the continental US. They are based on agricultural production and ancillary data from the US Census of agriculture, population data from the US census, and other sources. Full details on methodology and original sources can be found in the references below [1], [2], [3], [4], [5].
